# Adapting the SMART tube technology for flow cytometry in feline full blood samples

**DOI:** 10.3389/fvets.2024.1377414

**Published:** 2024-06-26

**Authors:** Katharina Zwicklbauer, Dominik von la Roche, Daniela Krentz, Laura Kolberg, Martin Alberer, Yury Zablotski, Katrin Hartmann, Ulrich von Both, Sonja Härtle

**Affiliations:** ^1^LMU Small Animal Clinic, Centre for Clinical Veterinary Medicine, LMU Munich, Munich, Germany; ^2^Department of Veterinary Sciences, AG Immunology, LMU Munich, Planegg, Germany; ^3^Division of Paediatric Infectious Diseases, Dr. von Hauner Children’s Hospital, University Hospital, LMU Munich, Munich, Germany; ^4^German Center for Infection Research (DZIF), Partner Site Munich, Munich, Germany

**Keywords:** flow cytometry, smart tube system (SMT), feline, full blood sample, long-term storage

## Abstract

Flow cytometry of blood samples is a very valuable clinical and research tool to monitor the immune response in human patients. Furthermore, it has been successfully applied in cats, such as for infections with feline immune deficiency virus (FIV). However, if cells are not isolated and frozen, analysis of anticoagulated blood samples requires mostly prompt processing following blood collection, making later analysis of stored full blood samples obtained in clinical studies often impossible. The SMART Tube system (SMART TUBE Inc., California, United States; SMT) allows fixation and long-term preservation of whole blood samples at −80°C. However, this system has so far only been applied to human biological samples. In the present study, a new flow cytometry SMART Tube protocol adapted for feline whole blood samples was successfully established allowing quantification of T-helper cells, cytotoxic T-cells, B-cells, monocytes, and neutrophils up to 2 years post sampling. Results obtained from frozen stabilized and fresh blood samples were compared for validation purposes and correlated to differential blood counts from a conventional hematology analyzer. Clinical applicability of the new technique was verified by using samples from a treatment study for feline infectious peritonitis (FIP). Using the new SMT protocol on retained samples, it could be demonstrated that long-term storage of these SMT tubes is also possible. In summary, the newly adapted SMT protocol proved suitable for performing flow cytometry analysis on stored feline whole blood samples, thus opening up new avenues for veterinary research on a variety of aspects of clinical interest.

## 1 Introduction

Flow cytometry is a very sophisticated and highly developed technique for analyzing the qualitative and quantitative characteristics of individual whole cells and cellular components (1). The physical properties (e.g., size, complexity/granularity, membrane integrity) and the expression of specific molecules (e.g., antigens on or in the cell) can be recorded quickly and simultaneously for each individual cell. Cells, therefore, can be divided into different populations and subpopulations. Flow cytometry is often used to characterize diseases in clinical settings in order to monitor the immune response in human patients (2, 3). The multiparametric, quantitative analysis also makes flow cytometry a powerful tool in biological sciences. Peripheral blood, bone marrow and lymph node aspirates, and cerebrospinal fluid are among the multitude of specimens that can be analyzed. Clinical flow cytometry in cats is currently primarily used for diagnosis and prognosis of hematopoietic neoplasms (lymphoma and leukemia) (4). It also has been successfully applied for infections with feline immune deficiency virus (FIV), in order to analyze diagnostic approaches or to monitor the immune responses in these cats (5).

However, only viable cells should be subjected to the initial staining process (6). For peripheral whole blood samples collected in ethylenediaminetetraacetic acid (EDTA)-anticoagulated tubes, cell stability has been demonstrated for a maximum of 48 h after collection (7) with only mild changes in leukocytes, lymphocytes and neutrophils, but in some cases significant changes in monocytes when measuring with automatic analyzers (8). This leaves only a very small time-window for analysis, especially for samples from clinical trials; this window is often too short, as in-house devices performing flow cytometry are rarely available, and samples from different sites often have to be shipped and stored before analysis. Although it is possible to isolate feline peripheral blood mononuclear cells (PBMC) using density gradient centrifugation and use traditional cryopreservation protocols to allow subsequent analysis by flow cytometry, a comparably large amount of blood is necessary to extract enough cells for conservation. In addition, isolation and freezing can alter cellular composition and impact the expression of certain markers.

The SMART Tube system (SMART TUBE Inc., California, United States; SMT) was developed for fixation and long-term storage of whole blood samples. This system has already been used in some mass and flow cytometry studies, but so far, only on human biological samples (9–12). An important benefit of whole blood storage and staining is the requirement for significantly smaller blood volumes compared to volumes needed for classical isolation techniques. This advantage is especially important in studies with longitudinal sampling in smaller animals such as cats, where the possible volume for blood withdrawal is severely restricted.

Therefore, the aim of this study was to establish a protocol for routine use of the SMT system with feline full blood samples and apply this protocol to samples of a clinical treatment study for cats with feline infectious peritonitis (FIP) after long-term storage.

## 2 Materials and methods

### 2.1 Sampling of feline blood

Spare EDTA-anticoagulated samples from peripheral whole blood of 20 healthy cats were collected at the LMU Small Animal Clinic in Munich. For fresh blood (FB) analysis, EDTA samples were stored at 4°C overnight and processed the next morning. For stored samples, 200 μL of EDTA whole blood was fixed and stabilized within a few hours after collection by mixing it with 270 μL of Proteomic Stabilizer Prot1 (SMART TUBE Inc., California, United States) in cryovials [micro screw-in tube 2 mL (Sarstedt AG & Co. KG, Nümbrecht, Germany)] according to the manufacturer’s instructions. After incubation for 10 min at room temperature (RT) the samples were immediately transferred to −80°C. The individual storage periods (at −80°C) for all SMT blood samples are shown in Supplementary Table S1.

### 2.2 Automatic hematologic analyzer for feline blood

Hematology examination from all collected fresh-blood EDTA samples of the healthy cats was performed at the LMU Small Animal Clinic in Munich using the in-house automatic analyzer ProCyte Dx (IDEXX Laboratories, Inc., Maine, United States). Hematology examination from samples of cats with FIP was performed using the automatic analyzer Cell-Dyn 3,500 (Abott Laboratories, IL, United States). When an invalid separation of leukocyte populations was present, additional microscopical examination of blood smears (for the validation study and the clinical application) was performed.

### 2.3 Antibodies

For flow cytometry, seven commercially available monoclonal antibodies (mAbs) were tested: anti-cat CD4- Fluorescein isothiocyanate (FITC) (clone 3-4F4) (SouthernBiotech, Birmingham, United States), anti-cat CD4-FITC (clone vpg34) (BioRad Laboratories, Feldkirchen, Germany), anti-cat CD8-PE (clone fCD8) (SouthernBiotech), anti-dog CD21 (clone CA2.1D6) (BioRad Laboratories), anti-human CD21-APC (clone B-ly4) (BD PharmingenTM, Heidelberg, Germany), anti-human CD14-PacificBlue (clone TÜK14) (BioRad Laboratories), and anti-cat MHCII (clone PF6J-6D) (BioRad Laboratories). Anti-cat MHCII was only available purified and was therefore conjugated to CF405M using the Mix-n-StainTM CFTM405M Antibody Labeling Kit (Sigma-Aldrich^®^, St. Louis, United States). Purified anti-dog CD21 was conjugated to PerCP-Cy5.5 using the Lynx Rapid Antibody Conjugation Kit (BioRad Laboratories) according to the manufacturer’s instructions. All mAbs were titrated prior to the start of the experiment for optimal working dilutions (see Table 1).

**Table 1:** Commercially available antibodies for cats and cross-reactive antibodies from other species (dog and human) tested for functionality for Smart Tubes with flow cytometry.

Antibody	Clone	Fluorochrome	Target cells	Reference	Dilution	SMART Tube
Mouse anti-cat CD4-FITC	3-4F4	FITC	T-helper cells	Ackley et al. (13)	1:50	no
Mouse anti-cat CD4-FITC	vpg34	FITC	T-helper cells	Callanan et al. (14)	1:40	yes
Mouse anti-feline CD8-PE	fCD8	PE	cytotoxic T-cells	Klotz and Cooper (15)	1:100	yes
Mouse anti-human CD14-PacificBlue	TÜK14	PacificBlue	monocytes, granulocytes	Jacobsen et al. (16)	1:50	no
Mouse anti-cat MHCII	PF6J-6D	CF405M^*^	lymphocytes, monocytes	Hunt et al. (17)	1:20	yes
Mouse anti-human CD21-APC	B-ly4	APC	B-cells	Fischer et al. (18)	1:5	yes
Mouse anti-dog CD21	CA2.1D6	PerCP-Cy5.5^#^	B-cells	Cobbold and Metcalfe (19)	1:500	no

no, antibody did work on fresh blood cells but not on the SMT-fixed cells; yes, antibody did work on both, the fresh blood cells and the SMT-fixed cells. ^*^This antibody is only available purified; so, it was conjugated to CF405M using the Mix-n-StainTM CFTM405M Antibody Labeling Kit (Sigma-Aldrich^®^, St. Louis, United States). ^#^This antibody was obtained purified; so, it was conjugated to PerCP-Cy5.5 using the Lynx Rapid Antibody Conjugation Kit (BioRad Laboratories, Feldkirchen, Germany).

### 2.4 Sample processing for flow cytometry

For flow cytometric analysis of FB samples, 100 μL blood was mixed with 2 mL of 10X RBC Lysis Buffer (ThermoFisher Scientific, Massachusetts, United States) and incubated for 15 min at RT in the dark. After centrifugation at 550 × g for 5 min at 18°C, the supernatant was discarded and the cell pellet was resuspended in 500 μL of staining buffer (PBS pH 7.2, 1% BSA, 0.1% NaN3).

To thaw the stored tubes, the manufacturers protocol was modified as follows: the tubes were thawed in a cold-water bath (10°C to 15°C) for 5 min. Thawed content was transferred to a new tube, mixed with 2 mL of 1X Thaw-Lyse Buffer (SMART TUBE Inc.) and incubated for 10 min at RT under constant rotation. After this, leukocytes were pelleted at 560 × g for 5 min at RT. The pellet was resuspended with 3 mL of 1X Thaw-Lyse Buffer and incubated at RT for 10 min under rotation. After another centrifugation at 560 × g for 5 min at RT the cell pellet was resuspended with 500 μL of staining buffer (PBS pH 7.2, 1% BSA, 0.1% NaN3).

From both fresh and stored samples, 1 ×10^5^ cells were transferred into a 96-well plate and incubated with 50 μL of the mAbs mixture (each antibody was titrated individually for optimal staining to generate a final panel in section 3.1; also, see Table 1) for 20 min in the dark on ice, followed by washing with 100 μL staining buffer and centrifugation at 725 × g for 1 min at 18°C. Pellets were resuspended in 400 μL staining buffer, transferred to a new tube and analyzed by flow cytometry. Due to the presence of a fixative in SMT tubes, which alters cell membrane integrity and precludes the use of viability dyes, we refrained from employing viability dyes for any of the samples to maintain consistency in treatment.

Flow cytometry was performed on a BD FACS Canto II Flow Cytometer (Becton Dickinson, Heidelberg, Germany). For each sample, 10,000 single cells were acquired. BD FACS DIVA and FlowJo (Tree Star Inc., OR, United States) software were used for data analysis.

The absolute numbers of each leukocyte subset were calculated as previously published (20): to obtain absolute cell numbers from flow cytometry samples, the determined percentage of the respective cell population was multiplied by the hematology analyzers leukocyte count. Lymphocyte numbers were calculated by adding up the numbers of CD4+, CD8+, CD21+, and marker negative/MHCII+/small cells.

### 2.5 Analyzing intra-assay precision and accuracy of the feline SMT protocol

To determine intra-assay precision for the cell numbers obtained by the new feline SMT protocol, a blood sample of one healthy cat was split into five FB and five SMT samples, which were frozen afterwards and analyzed by flow cytometry processing. Coefficients of variation (CVs, in percent) were calculated as standard deviation (SD)/mean × 100.

To test for accuracy of the newly established feline SMT protocol, blood samples of 20 cats were split into triplicates and subsequently analyzed with conventional automated hematology analyzer (Diff) and by flow cytometry on either FB or SMT-fixed samples to compare numbers of lymphocytes, monocytes, and neutrophils. The cell numbers were calculated as described above.

### 2.6 Clinical application of the established protocol to cats from a FIP treatment study

In a recent clinical treatment study, five cats suffering from FIP received orally for 84 days the multi component drug Xraphconn^®^ (Mutian Life Sciences Limited, Nantong, China) containing the nucleoside analogue GS-441524 as previously published (21). During this treatment study, EDTA samples were collected with the SMT system according to the manufacturer’s instructions at six different time points from day 0 (before treatment initiation) through days 7, 14, 28, 56, and 83 (last day of treatment). In addition, EDTA samples of five healthy cats were collected with the SMT system. These cats were anti-feline coronavirus (FCoV) antibody-negative in serum and had no fecal FCoV shedding, both of which was determined as previously described (21–23).The SMTs of healthy and diseased cats were thawed and processed as described above. Storage times are given in Supplementary Table S1.

### 2.7 Statistical analysis

For a first comparison of Diff, FB, and SMT, Box plots were visualized for distribution of the data using MS Excel (version 2313). Data were analyzed using R statistical language (version 4.0.3; R Core Team, 2020). Pearson’s correlation coefficient [with the rules tiny to small correlation *r* < 0.2, medium correlation *r* < 0.3, large correlation *r* < 0.4, and very large correlation *r* ≥ 0.4 (24)], Bland–Altman plots depicting the mean bias ± 2 SD, and results of a Passing-Bablok regression analysis were reported.

## 3 Results

### 3.1 Antibody panel

Main objective of the used staining panel was to discriminate the different leukocyte subsets and, in addition to classical hematologic analysis, to address different lymphocyte subsets. Therefore, commercially available mAbs were tested for their functionality on SMT samples (see Table 1). Several mAbs resulted in a good staining pattern on FB. However, they did not work on SMT samples [anti-CD4-FITC (clone 3-4F4), anti-CD21-PerCP-Cy5.5 (clone CA2.1D6)], most likely due to the fixation process of the SMT system (see Supplementary Figures S1A,B). The anti-CD14 mAb showed good results on both FB and SMT samples but stained monocytes and neutrophils equally, so the desired differentiation of monocytes within the population of large cells was not possible (see Supplementary Figure S1C).

Four of the tested mAbs, either cat-specific or described as cross-reactive, gave good results likewise on FB and SMT samples and stained the intended cell populations (see Supplementary Figure S2). Hence, the final flow cytometry protocol included the following mAbs: anti-cat-CD4- FITC (vpg34) to stain T-helper cells, anti-cat-CD8-PE (fCD8) for cytotoxic T cells, anti-human-CD21-APC (B-ly4) for B cells, and anti-cat-MHCII-PacificBlue (PF6J-6D) to discriminate monocytes from neutrophils.

Although for all mAbs, mean fluorescence intensity (MFI) was significantly lower on cells from SMT than FB samples (40–45% for CD4, CD21 and MHCII, 25% for CD8), clear discrimination of even the most affected dim target populations was still possible (Supplementary Figure S2).

### 3.2 Gating strategy for flow cytometry

**Figure 1:**
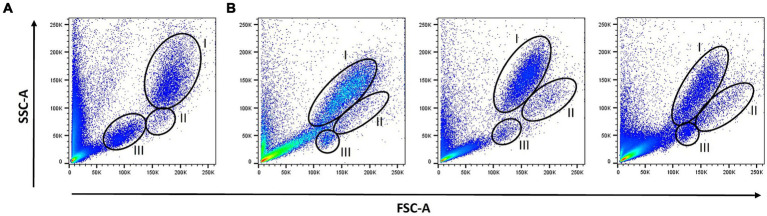
Scatter profiles: Forward scatter/Side scatter (FSC /SSC) profiles of lyzed fresh blood **(A)** and lyzed SMART Tube (SMT)-fixed blood samples from three different cats **(B)** were determined by flow cytometry. The different leukocyte populations were gated according to their FSC/SSC scatter profiles: neutrophils (I), monocytes (II), and lymphocytes (III).

Strikingly, flow cytometric analysis of SMT samples revealed FSC/SSC scatter plots, which differed considerably from that of FB and between individual cats. While discrimination between neutrophils and lymphocytes became more difficult after fixation, visibility of the otherwise hardly distinguishable monocyte population was often improved (Figure 1).

**Figure 2:**
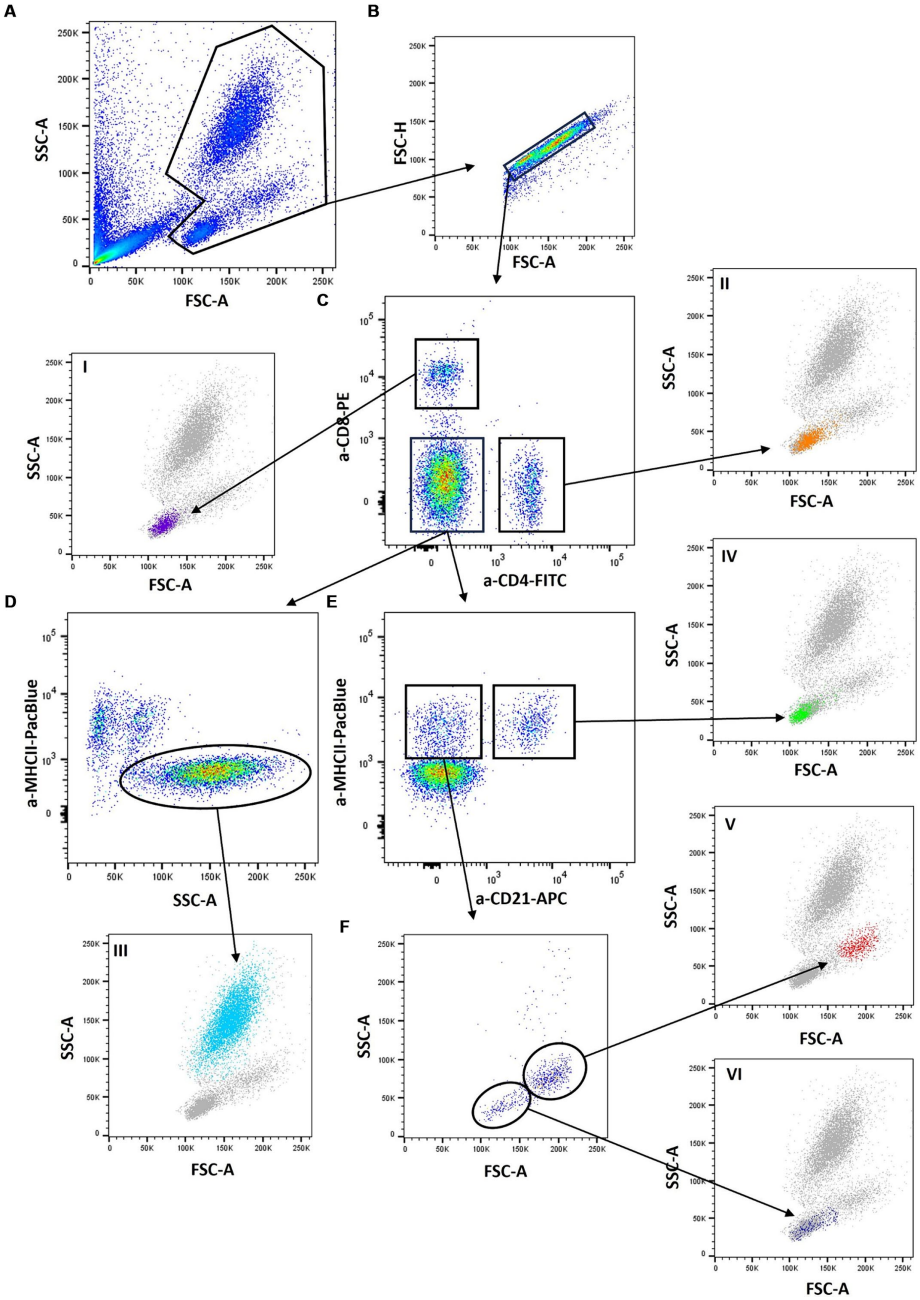
Gating strategy: A lyzed SMART Tube (SMT) blood sample was stained with anti-cat-CD4- FITC (vpg34), anti-cat-CD8-PE (fCD8), anti-huCD21-APC (B-ly4), and anti-cat-MHCII-PacificBlue (PF6J-6D) and analyzed by flow cytometry. First, feline leukocytes were identified by their Forward scatter/Side scatter (FSC /SSC) profiles **(A)**, followed by doublet exclusion **(B)**. CD4 and CD8 were used to address CD4+ helper T cells and CD8+ cytotoxic T cells. Double-positive cells were not observed (**C**, I and II). From the CD4/CD8 double-negative population neutrophils were identified through high SSC and a negative MHCII staining (**D**, III). B cells and monocytes were addressed in a CD21/MHCII plot **(E)** with CD21+/MHCII+ B cells and a CD21-/MHCII+ cell population. When the latter was further examined according to its FSC/SSC scatter characteristics **(F)**, two distinct populations could be identified: a more homogeneous population of large cells, which we regard as monocytes (V) and an additional population of smaller cells. Since these CD4-/CD8-/CD21-/MHCII+ cells have a lymphocyte scatter profile (VI), they were regarded as “marker negative lymphocytes”.

To address the different subpopulations, the following gating strategy was applied: First, feline leukocytes were identified by their FSC/SSC scatter profiles (Figure 2A), followed by doublet exclusion (Figure 2B). CD4 and CD8 were used to address CD4+ helper T cells and CD8+ cytotoxic T cells. Double positive cells were not observed (Figure 2C, I and II).

From the CD4/CD8 double-negative population neutrophils were identified through high SSC and a negative MHCII staining (Figure 2D, III). B cells and monocytes were addressed in a CD21/MHCII plot (Figure 2E) with CD21+/MHCII+ B cells and a CD21-/MHCII+ cell population. When the latter was further examined according to its FSC/SSC scatter characteristics (Figure 2F), two distinct populations could be identified: a more homogeneous population of large cells, which we regarded as monocytes (Figure 2F, V) and an additional population of smaller cells. Since these CD4-/CD8-/CD21-/MHCII+ cells have a lymphocyte scatter profile (Figure 2F, VI), they were regarded as “marker-negative lymphocytes.” As no natural killer (NK) cell marker was included in the applied antibody panel, a proportion of those “unstained” lymphocytes were supposedly NK cells but other lymphocyte populations could equally contribute.

### 3.3 Validation of the protocol

In order to analyze intra-assay precision of the applied protocol, one blood sample of a healthy cat was split into five FB and five SMT samples and analyzed by flow cytometry. As shown in Table 2, SD for all cell populations was very low (0.01–0.02 ×10^9^ c/L) and CV of intra-assay precision was below 1% for all cell types (Table 2).

**Table 2:** Intra-assay precision estimated from 5 fresh blood and 5 fixed aliquots from a blood sample of a single cat analyzed by flow cytometry.

	Neutrophils	Monocytes	CD4+	CD8+	CD21+
Samples	Mean ± SD (CV in %)	Mean ± SD (CV in %)	Mean ± SD (CV in %)	Mean ± SD (CV in %)	Mean ± SD (CV in %)
Fresh blood	2.93 ± 0.02 (0.62)	0.29 ± 0.00 (0.84)	0.91 ± 0.01 (0.61)	1.03 ± 0.01 (0.73)	0.92 ± 0.01 (0.60)
Smart tube	3.34 ± 0.01 (0.26)	0.25 ± 0.00 (0.75)	0.73 ± 0.00 (0.65)	0.84 ± 0.00 (0.43)	0.75 ± 0.00 (0.48)

SD, standard deviation; CV, coefficient of variation. The absolute leukocyte counts determined by routine hematologic procedures using the in-house automatic analyzer ProCyte Dx (IDEXX Laboratories, Inc., Maine, United States) were multiplied by the percentage of the respected subpopulations from total single cells determined by flow cytometry; all means of the cell counts of each subpopulation are given in x109/l.

For accuracy of the newly established feline SMT protocol, the numbers of lymphocytes, monocytes, and neutrophils analyzed with Diff, FB and SMT were compared to each other.

**Figure 3:**
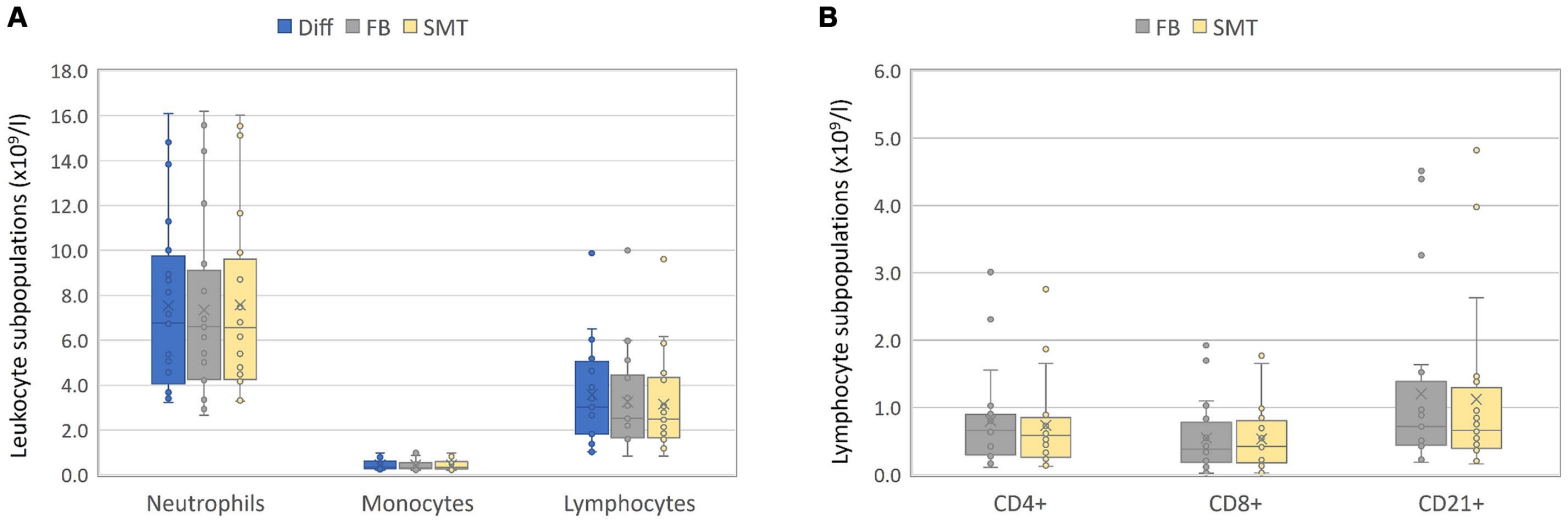
Validation – absolute counts: Blood of 20 healthy cats was split in three and objected to automated hematology analysis (Diff), analyzed by flow cytometry within 24 h (FB) or fixed/frozen in a SMART Tube (SMT) before flow cytometric analysis. For FB and SMT samples, the absolute numbers of each leukocyte subset were calculated by multiplication of Diff. Absolute blood cell counts from all three techniques for monocytes, lymphocytes, and neutrophils are presented in **(A)**; lymphocyte subpopulations obtained by flow cytometry are shown in **(B)**. Box plots include the second and the third quantile, whiskers include all values within the 1.5 interquartile range, dots outside the box represent outliers, dots inside the box the values; the horizontal line represents the median, the mean is presented as a cross.

**Figure 4:**
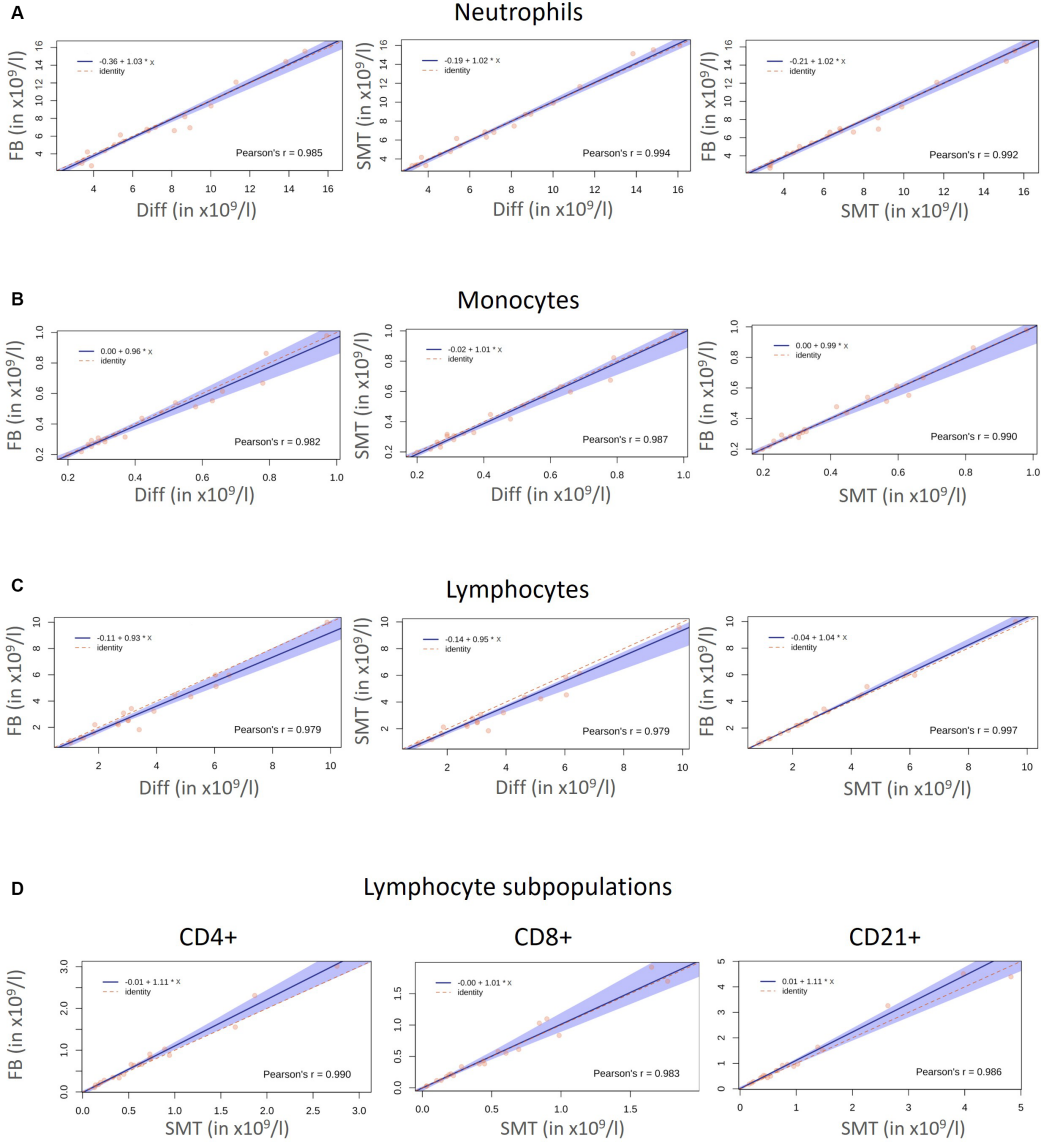
Correlation Diff/FB/SMT: Correlation and agreement between the absolute cell numbers (x10^9^/l) for neutrophils **(A)**, monocytes **(B)**, and lymphocytes **(C)** of 20 healthy cats obtained with an automated hematology analyzer (Diff), by flow cytometry within 24 h (FB) and with fixed/frozen Smart Tubes (SMTs). Side scatter diagrams with the red dots represent measured values. The solid blue line represents the regression line, the dashed red line represents the line of identity, and 95% confidence intervals (CI) are represented by the blue shaded area. Comparison for lymphocyte subpopulations obtained by flow cytometry with FB and SMT are shown in **(D)**. Pearson’s correlation coefficient (r) is given in each diagram.

In general, a first juxtaposition of cell numbers revealed similar mean cell counts for all cell populations although flow cytometry-based analysis resulted in slightly lower cell counts, which was most pronounced for lymphocytes [mean Diff 3.6 ×10^9^ c/L vs. 3.2 ×10^9^ c/L (FB and SMT) (Figure 3A; Supplementary Table S2)]. The correlation between the different methods was very large for all cell populations with Pearson’s r ranging from 0.979 to 0.997. Results of Passing–Bablok regression showed slopes for all comparisons between 0.80–1.11. All 95% coefficients of variation (CIs) included 1.00 indicating that there was no proportional bias between the methods for any of the cell populations tested. All intercepts included 0.0 within the 95% CIs (ranging from −0.91–0.23) indicating that there was no significant constant error between the methods for any cell count (Figures 4A–C and Table 3). The Bland–Altman plots showed that all but a few values were within the limits of agreement (LoA), and only a single value when comparing neutrophil counts between SMT and FB, was outside the 95% CI of the upper LoA (Supplementary Figures S3A–C).

**Table 3:** Passing-Bablok [with 95% confidence intervals (CI)] regression analysis between the different methods for neutrophils, monocytes, and lymphocytes and Pearson’s correlation coefficient (r).

	Neutrophils r Slope (CI)/ Intercept (CI)	Monocytes r Slope (CI)/ Intercept (CI)	Lymphocytes^*^ r Slope (CI)/ Intercept (CI)	CD4+ r Slope (CI)/ Intercept (CI)	CD8+ r Slope (CI)/ Intercept (CI)	CD21+ r Slope (CI)/ Intercept (CI)
FB/ Diff	0.985 1.03 (0.94–1.11)/ −0.36 (−0.91–0.23)	0.982 0.96 (0.81–1.10)/ 0.00 (−0.04–0.05)	0.979 0.93 (0.84–1.04)/ −0.11 (−0.47–0.08)	–	–	–
SMT/Diff	0.994 1.02 (0.97–1.09)/ −0.19 (−0.72–0.11)	0.987 1.01 (0.85–1.08)/ −0.01 (−0.05–0.04)	0.979 0.95 (0.80–1.01)/ −0.14 (−0.41–0.12)	–	–	–
SMT/ FB	0.992 1.02 (0.95–1.08)/ −0.21 (−0.66–0.18)	0.990 1.00 (0.84–1.05)/ 0.00 (−0.02–0.05)	0.997 1.04 (1.01–1.11)/ −0.04 (−0.16–0.01)	0.990 1.11 (0.99–1.25)/ −0.01 (−0.09–0.03)	0.983 1.01 (0.88–1.23)/ −0.00 (−0.05–0.02)	0.986 1.11 (0.91–1.20)/ 0.00 (−0.04–0.08)

r, Pearson’s correlation coefficient; CI, 95% confidence intervals; SMT, fixed cells with Smart Tube System; Diff, differential blood count with automatic hemocytometer, FB, fresh blood. ^*^Lymphocyte numbers were calculated by adding up the numbers of CD4+, CD8+, CD21+ and “marker-negative” lymphocytes.

In detail, comparison of neutrophil and monocyte counts revealed Pearson’s r 0.985 ≥ r ≤ 0.994 (neutrophils) and 0.982 ≥ r ≤ 0.990 (monocytes) showing no significant biases between all methods (Figures 4A,B and Supplementary Figures S3A,B). As already assumed from the mean cell counts, the comparison between lymphocyte counts from the different methods revealed the lowest correlation coefficient with *r* = 0.979 (Diff vs. FB/SMT), which, however, still indicates a very large correlation; the biases were significant at 0.33 and 0.43 ×10^9^ c/L (differences ranging from −0.32–1.58 ×10^9^ c/L), and with the values plotted particularly above the 0 line, it is again indicated that Diff values tended to be higher than FB/SMT. For FB vs. SMT we found *r* = 0.997 with a bias of only −0.1 ×10^9^ c/L and differences ranging from −0.58–0.19 ×10^9^ c/L (Figure 4C and Supplementary Figure S3C). Since discrimination of lymphocyte subpopulations is not possible using Diffs, CD4+, CD8+ and CD21+ cell counts were only compared between FB and SMT samples (Figure 3B). Here, too, the 95% CI of all slopes and intercepts included 1.00 and 0.00 indicating no proportional bias or constant error. For the three lymphocyte subpopulations, the direct comparison between FB and SMT cells revealed a very large correlation with *r* ≥ 0.983. With a few exceptions, the values were within the upper and lower LoA; comparing SMT vs. FB for CD4+, one value was without the 95% CI of the lower LoA. Biases were between −0.08 and − 0.01 ×10^9^ c/L (differences ranging from −0.63–0.42 ×10^9^ c/L), with minimal significance for CD4+ (Figure 4D; Supplementary Figure S3D and Table 3). Overall, with the anticipated exception of lymphocyte numbers, comparison between Diff counts and flow cytometry of both FB and fixed, frozen SMT samples revealed high correlation and agreement between methods. Comparison of lymphocyte subpopulations between FB and SMT samples showed only minimal differences, demonstrating that fixation and storage of blood samples in SMT tubes is possible.

### 3.4 Application to long-term stored clinical samples

Finally, we tested the applicability of our protocol for clinical samples using samples from an earlier FIP treatment study. Feline infectious peritonitis (FIP) is a fatal feline coronavirus (FCoV)-induced and immunologically mediated disease characterized by systemic granulomatous vasculitis and perivasculitis (25). Treatment of FIP diseased cats with oral GS-441524 led to a complete recovery of cats with a significant improvement of most clinicopathological parameters [hematological and clinical chemistry parameters including the acute phase protein serum amyloid A (SAA)], which can be highly elevated in cats with FIP during the initial 14 days of treatment (21). Throughout the study, SMT blood samples were obtained and stored at −80°C. The mean sample storage time was 2 years (plus/minus a few weeks, depending on when the cats were included into the study, see Supplementary Table S1 for detailed information). We analyzed SMT samples from five healthy cats (not infected with FCoV) and five cats with FIP on days 0, 7, 14, 28, 56, and 83 after treatment initiation and compared the obtained results with Diff performed on the day of sampling.

**Figure 5:**
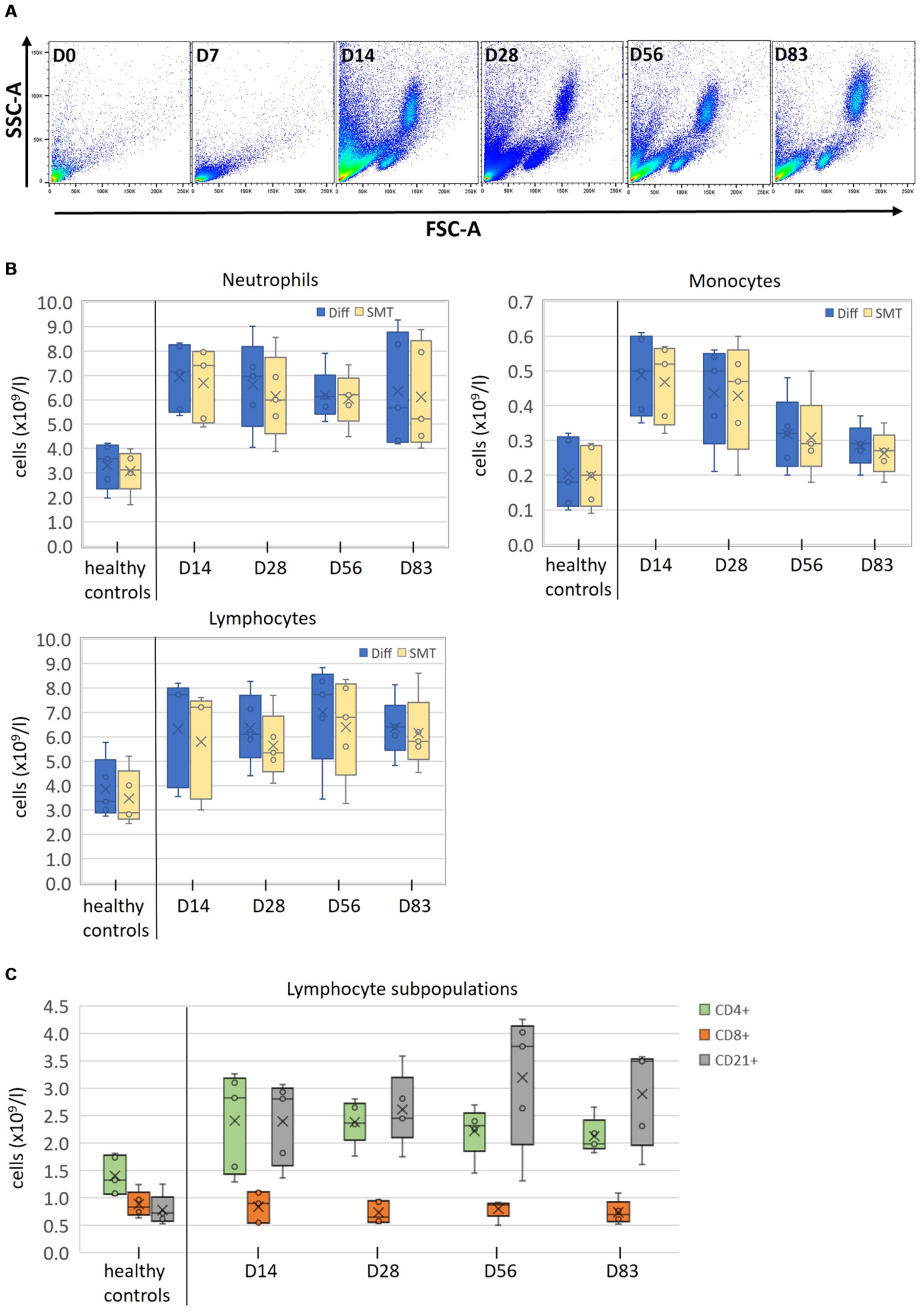
Measurement of clinical SMT samples after long time storage **(A)** FSC/SSC profiles of SMT blood samples from one representative cat suffering from feline infectious peritonitis (FIP) before (D0), and during (D7, 14, 28, 56, and 83) antiviral treatment. **(B)** Absolute counts of all cell populations (neutrophils, monocytes, and lymphocytes) from five healthy control cats collected at a single timepoint and from five FIP cats at the indicated timepoints, analyzed with an automated hematology analysis at the day of sampling (Diff; blue Box plots) or by flow cytometry with fixed/frozen SMART Tubes (SMT; orange Box plots) up to 2 years post sampling. **(C)** Lymphocyte subpopulations (CD4+, CD8+, and CD21+) of indicated SMT samples after long time storage. Box plots include the second and the third quantile, whiskers include all values within the 1.5 interquartile range, dots outside the box represent outliers, dots inside the box the values; the horizontal line represents the median, the mean is presented as a cross.

Strikingly, the thawing process of SMT samples from the FIP-diseased cats did not work successfully for any sample from days 0 and 7. Samples appeared visibly clotted and, after thawing and lysis, contained large amounts of debris from which no intact cells could be isolated (see Figure 5A, D0 + D7 for representative scatter plots). Accordingly, no comparison between Diff and flow cytometry could be made for D0 and D7 (Figure 5B).

In contrast, control samples and samples from days 28, 56, and 83 could be thawed and processed without any problems, including samples with even longer storage times than D0 and D7 (see Figure 5A, D14–D83 and Supplementary Table S1). On day 14, samples of three cats showed minor signs of clotting, which, however, did not affect further processing.

Owing to the limited sample size, a comprehensive statistical analysis was not conducted. Mean and distribution for neutrophils and monocytes was similar between Diff and SMT, while values for lymphocyte numbers obtained for SMT samples were consistently lower than those from the initial Diff (Figure 5B and Supplementary Table S3).

Importantly, it was possible to determine the number of lymphocyte subpopulations in all non-clotted samples, which revealed a largely identical number of CD8+ cells between control cats and the treatment group and considerable differences for CD4+ and CD21+ cells (Figure 5C).

## 4 Discussion

On-site sample processing during clinical trials is challenging. However, until now, EDTA samples from cats and other species had to be processed within a maximum of 48 h (7). The SMT system for whole blood was developed to allow for fixation and conservation at −80°C for a long period, but this technique had so far been only applied to human biological samples. In this study, the SMT protocol was successfully adapted for feline blood samples. A new flow cytometry protocol to address leukocytes, monocytes, neutrophils, and lymphocytes was established accurately separating lymphocytes into CD4+ T-helper cells, CD8+ cytotoxic T cells and CD21+ B cells even after prolonged storage of samples.

Only commercially available mAbs were used in the staining panel aiming for a wide availability. As the range of cat-specific and cross-reactive commercially available mAbs is currently very limited, while highly desirable to identify all T cells, no surface anti-CD3 or anti-TCR staining could be included. Though a feline CD3 monoclonal antibody recognizing a surface epitope has been described (26) this antibody is not commercially available and has so far rarely been used due to difficulties with conjugation. The anti-human CD3 monoclonal antibody clone CD3-12, targeting an intracytoplasmic epitope of CD3e and cross-reactive with CD3 molecules of multiple species, has been used and reported with success for the detection of feline T cells, and is commercially available conjugated to several different fluorochromes (27). However, this antibody requires permeabilization for intracytoplasmic staining, a step which we did not want to include in our initial protocol. But that could be an option for its further refinement. As potential alternative, staining for CD5 expression was reported as option to address all feline T cells (28) and several mAbs (f43; FE1.1B11) are commercially available. Both should be tested in future for their suitability in staining SMT-fixed cells.

From the tested mAbs, the potential monocyte marker anti-CD14 had to be omitted eventually as it did not show the desired staining specificity but instead resulted in an undistinguishable staining of monocytes and neutrophils. Some mAbs (an anti-CD4 and an anti-CD21 clone) worked well on FB but staining patterns were altered by fixation to a great extent, a well-known phenomenon, as fixation can affect cell membrane permeability leading to unspecific staining or disrupts the antibody’s epitopes leading to loss of staining (29, 30).

Finally, four mAbs specific for CD4, CD8, CD21 and MHC class II were identified showing excellent performance on both FB and SMT samples. Staining for CD4 and CD8 to address T-helper cells and cytotoxic T cells is common practice in cats (31–33). CD4 and CD8 expression on feline PBMC CD3+ cells are mutually exclusive and hardly any double positive cells were found in the present study as well as in previous studies (34). Still, it must be considered that in many species, e.g., dogs and pigs (35, 36), CD8 is also expressed on a subset of γδ T cells and in some species additional CD8 is found on NK cells (37–40). Due to the lack of feline TCR-specific mAbs to discriminate between αβ and γδ T cells and commercially available surface markers for NK cells, we could not address these cells. However, in dogs, a related species, with more available markers, only 0.5–3% of peripheral blood lymphocytes are CD8 expressing γδ T cells CD8 (41). Assuming that cats are also a γδ-low species and considering that about 15% of lymphocytes were CD8+, the content of CD8+ γδ T cells is probably only marginal. In addition, Vermeulen and colleagues showed, that feline NK cells represent only about 1–5% of PBL and only 10% of these cells express CD8, resulting in only 0.1–0.5% of CD8 expressing non-T cells (42). Thus, despite some limitations it seems justified to refer to the gated CD8+ cells as cytotoxic T cells. Unfortunately, the lack of CD3 staining prevents the identification of a potential small CD4-/CD8- double negative T cell population which was reported in other species (35).

CD21 is an established B cell marker in many species (19, 43, 44) and although the cat-specific anti-CD21 mAb did not work on SMT, the cross-reactive human clone with identical staining on FB could be included into the antibody panel. Nevertheless, as in other species, anti-CD21 staining probably does not address all stages of feline B cell differentiation. In other species like humans, B cells lose CD21 expression when they differentiate into plasmablasts (45) and the presence of some CD21−/immunoglobulin+ B cells, which is larger in cell size (FSC) and probably resembles plasmablasts was reported in feline blood (46). Activated memory B cells in humans and non-human primates also lose CD21 expression (47). It is therefore quite possible that CD21+ cells represent not all differentiation states of feline B cells and staining for the B cell receptor complex (e.g., with anti-CD79) might be an option to address further B cell subpopulations in the future.

However, it is highly likely that these CD21−B cell stages cells express MHCII and hence could be part of the MHCII+/marker-negative small cell population in the gating strategy.

In addition to B cells and monocytes, almost all other feline lymphocytes express MHC class II without prior activation, but neutrophils are MHC class II-negative (17). Hence, anti-MHCII staining were used to discriminate neutrophils and monocytes in the present study. Though comparison of monocyte numbers from an automated analyzer and the gating strategy of the SMT protocol showed a very large correlation, it must be mentioned that in many species monocytes express to a different extent CD4 (48). For example, in dogs next to the majority of MHCII+/CD4− monocytes two minor subsets of MHCII+/CD4+ and even MHCII−/CD4+ monocytes were identified (49). Hence, the small subset of larger cells in our CD4+ gate, instead being activated T helper cells, could also be monocytes. If antibodies are available, a possible solution to overcome this problem in the future studies would be the inclusion of a specific T cell or monocyte marker.

For validation of the used staining protocol with blood samples obtained from healthy cats, flow cytometry derived numbers of FB and SMT samples were compared to cell counts obtained by an automatic hematology analyzer. It is known that accuracy of automated leukocyte differentials particularly for animal species and especially the quantification of monocytes and basophils does not reach the accuracy of microscopic review. Though microscopic review of blood smears in this study was only performed when the automated differential was inconclusive, we have put up with this limitation as automated leukocyte counts were only used to verify agreement between methods and not accuracy of the real values.

The comparison revealed a very good agreement for the quantification of monocytes and neutrophils. Flow cytometry-based lymphocyte counts were generally lower than the automated counts, but still showed a very large correlation. A possible explanation for the reduced numbers could be that lymphocyte numbers were calculated as the sum of CD4+, CD8+, CD21+ and MHCII+/marker-negative/small cells, which does not include potential additional MHCII− lymphocytes, such as CD4−/CD8− T cells, NK cells and CD21− B cell subsets. In future studies, addition of an anti-CD18 mAb, which is reported as panleukocyte marker with good lymphocyte discrimination (50), might help to solve this issue, provided it works on fixed cells. However, considering a study evaluating the ProCyte Dx, one of the automated analyzers used in this study, the results showed good to excellent correlations for most different leukocyte counts, but deviations of up to 30% for lymphocyte counts (51). Therefore, results of the used flow cytometry protocol without anti-CD18 were satisfactory.

Comparison of lymphocyte subpopulations between FB and SMT samples showed a very large correlation and agreement with only minimal biases, demonstrating that fixation, freezing, and thawing and a second lysis step in the SMT protocol did not affect the number of T and B cells. Thus, the SMT technology can be successfully applied to feline blood samples. Though the validation was carried out on samples that had only been frozen for an average time of 2 days, the comparable results of the FB samples and the paired long-term SMT-fixed and frozen samples from FIP diseased (at the later study timepoints) and healthy control cats suggests that long-term preservation and processing is also possible with reliable results.

Availability of the SMT technology is of paramount importance for trial purposes, allowing for collection of biological samples during routine business and analysis of respective samples at a later time point. In addition, collection of samples can now be performed at various study site with sample analysis taking place in a different site or laboratory.

To assess applicability of the new feline SMT protocol in practice, SMT samples from cats in a FIP treatment study were investigated. Strikingly, in contrast to samples from healthy cats, not a single sample from a cat with FIP from day 0 to day 7 after treatment initiation could be analyzed. Regular thawing of SMTs was not possible, as the stabilized samples were visibly clotted and contained only large amounts of debris following lysis, and no intact cells could be isolated. Interestingly, one human study using SMTs for hospitalized COVID-19 patients reported similar results. The SMTs worked very well with healthy donor samples, but performance was poor with samples of a large number of acute COVID-19 patients. This was discussed to be related to polymerized fibrin or other plasma factors associated with COVID-19-associated coagulopathy (9). Cats with FIP can also develop disseminated intravascular coagulopathy due to activation of complement and clotting factors and marked vasculitis during the inflammatory process (52). Indeed, it is already known that inflammatory markers, such as SAA and alpha-1-acid glycoprotein (53), can be highly elevated in cats with FIP, which indicates a severe inflammatory response (54–57). Cats in the treatment study had very high SAA concentrations before and up to 7 days of treatment, which then decreased rapidly and were in the reference range by day 14 (21). The marked decrease of inflammatory markers in response to treatment indicates a strong attenuation of the hyperinflammatory FIP-mediated stage, and from the day of normalization of inflammatory markers on, SMT samples could be analyzed without problems. Although COVID-19 can also cause a strong inflammatory response in humans, affected SMT samples in the above-mentioned study were still processable with protocol modifications, which however, did not work with affected cat SMT samples of the present study. This might be explained by an even more pronounced inflammatory status in cats with FIP or an amplification of the effect by feline platelets, which, even in healthy cats, generally have a high tendency to clump after blood collection regardless of the collection technique (58).

Taken together, this first application of the newly established feline SMT flow cytometry protocol demonstrates a general possibility for flow cytometric analysis after long-term storage of full blood samples, and that in its current form can probably not be used for samples taken during a highly inflammatory state of a patient. Future experiments will need to address whether this phenomenon also occurs in other acute diseases such as septic conditions and severe bacterial infections or whether this is FIP-specific. In a next step it should be evaluated whether the sampling procedure can be modified for samples from such conditions.

In conclusion, in the present study we demonstrate for the first time that the SMT system is successfully applicable for feline full blood samples. Using our newly established protocol, samples can be collected, stabilized, sent to a laboratory for flow cytometric analysis, or stored to address later arising questions. In addition, the successful technology transfer from human to veterinary medicine will likely pave the way to its, albeit slightly modified, application in other animal species. This holds the potential to significantly improve and simplify workflows, subsequently enhancing the amount of knowledge that can be obtained from animal studies.

## Data availability statement

The original contributions presented in the study are included in the article/Supplementary material, further inquiries can be directed to the corresponding author.

## Ethics statement

The animal studies were approved by Government of Upper Bavaria, reference number 55.2-2532.Vet_02-20-52 and ethical committee (reference number 261-19-03-2021) of the Centre for Clinical Veterinary Medicine of the LMU Munich. The studies were conducted in accordance with the local legislation and institutional requirements. Written informed consent was obtained from the owners for the participation of their animals in this study.

## Author contributions

KZ: Conceptualization, Formal analysis, Investigation, Methodology, Resources, Validation, Visualization, Writing – original draft, Writing – review & editing. DR: Investigation, Methodology, Writing – review & editing. DK: Resources, Writing – review & editing. LK: Resources, Writing – review & editing. MA: Conceptualization, Writing – review & editing. YZ: Statistical Analysis, Writing – review & editing. KH: Conceptualization, Funding acquisition, Writing – review & editing. UB: Conceptualization, Funding acquisition, Writing – review & editing. SH: Conceptualization, Formal analysis, Funding acquisition, Methodology, Validation, Visualization, Writing – original draft, Writing – review & editing.

## References

[1] Herzenberg LA Parks D Sahaf B Perez O Roederer M Herzenberg LA. The history and future of the fluorescence activated cell sorter and flow cytometry: a view from Stanford. Clin Chem. (2002) 48:1819–27. doi: 10.1093/clinchem/48.10.1819, PMID: 12324512

[2] Bariogie B Raber MN Schumann J Johnson TS Drewinko B Swartzendruber DE. Flow cytometry in clinical cancer research. Cancer Res. (1983) 43:3982–97. 6347364

[3] Ibrahim SF Van Den Engh G. Flow cytometry and cell sorting. Adv Biochem Eng Biotechnol. (2007) 106:19–39. doi: 10.1007/10_2007_073 17728993

[4] Martini V Bernardi S Marelli P Cozzi M Comazzi S. Flow cytometry for feline lymphoma: a retrospective study regarding pre-analytical factors possibly affecting the quality of samples. J Feline Med Surg. (2018) 20:494–501. doi: 10.1177/1098612X17717175, PMID: 28675320 PMC11104065

[5] Murphy B Hillman C McDonnel S. Peripheral immunophenotype and viral promoter variants during the asymptomatic phase of feline immunodeficiency virus infection. Virus Res. (2014) 179:34–43. doi: 10.1016/j.virusres.2013.11.017, PMID: 24291288 PMC4038716

[6] Trewhitt KG. Bone marrow aspiration and biopsy: collection and interpretation. Oncol Nurs Forum. (2001) 28:1415. 11683311

[7] Davis BH Dasgupta A Kussick S Han JY Estrellado A. Validation of cell-based fluorescence assays: practice guidelines from the ICSH and ICCS – part II – preanalytical issues. Cytometry B Clin Cytom. (2013) 84:286–90. doi: 10.1002/cyto.b.21105, PMID: 24022851

[8] Granat F. Geffré A. Bourgès-Abella N., Braun J-P., Trumel C. Changes in haematology measurements with the Sysmex XT-2000iV during storage of feline blood sampled in EDTA or EDTA plus CTAD. J Feline Med Surg (2013) 15:433–444. doi: 10.1177/1098612X12469967, PMID: 23264612 PMC10816317

[9] Geanon D Lee B Gonzalez-Kozlova E Kelly G Handler D Upadhyaya B. A streamlined whole blood CyTOF workflow defines a circulating immune cell signature of COVID-19. Cytometry A. (2021) 99:446–61. doi: 10.1002/cyto.a.24317, PMID: 33496367 PMC8013522

[10] Chen L Youssef Y Robinson C Ernst GF Carson MY Young KA. CD56 expression marks human group 2 innate lymphoid cell divergence from a shared NK cell and group 3 innate lymphoid cell developmental pathway. Immunity. (2018) 49:464–476.e4. doi: 10.1016/j.immuni.2018.08.010, PMID: 30193847 PMC6148384

[11] Aghaeepour N Ganio EA Mcilwain D Tsai AS Tingle M van Gassen S. An immune clock of human pregnancy. Sci Immunol. (2017) 2:2. doi: 10.1126/sciimmunol.aan2946, PMID: 28864494 PMC5701281

[12] Spitzer MH Carmi Y Reticker-Flynn NE Kwek SS Madhireddy D Martins MM. Systemic immunity is required for effective cancer immunotherapy. Cell. (2017) 168:487–502.e15. doi: 10.1016/j.cell.2016.12.022, PMID: 28111070 PMC5312823

[13] Ackley CO Hoover LA Cooper MD. Identification of a CD4 homologue in the cat. Tissue Antigens. (1990) 35:92–8. doi: 10.1111/j.1399-0039.1990.tb01762.x, PMID: 2188396

[14] Callanan JJ Racz P Thompson H Jarrett O. Morphologic characterization of the lymph node changes in feline immunodeficiency virus infection as an animal model of AIDS1 In: Racz P Letvin NL Gluckman JC, editors. Animal Models of HIV and Other Retroviral Infections (1993). (Basel, CHE: S.Karger AG), 115–36.

[15] Klotz FW Cooper MD. A feline thymocyte antigen defined by a monoclonal antibody (FT2) identifies a subpopulation of non-helper cells capable of specific cytotoxicity. J Immunol. (1986) 136:2510–4. doi: 10.4049/jimmunol.136.7.2510, PMID: 3081644

[16] Jacobsen CN Aasted B Broe MK Petersen JL. Reactivities of 20 anti-human monoclonal antibodies with leucocytes from ten different animal species. Vet Immunol Immunopathol. (1993) 39:461–6. doi: 10.1016/0165-2427(93)90075-F, PMID: 8116221

[17] Hunt P Mcconnell I Grant CK Else RW Hopkins J. Variable expression of major histocompatibility complex class II in the domestic cat. Res Vet Sci. (1995) 59:195–200. doi: 10.1016/0034-5288(95)90001-2, PMID: 8588090

[18] Fischer E Delibrias C Kazatchkine MD. Expression of CR2 (the C3dg/EBV receptor, CD21) on normal human peripheral blood T lymphocytes. J Immunol. (1991) 146:865–9. doi: 10.4049/jimmunol.146.3.865, PMID: 1703182

[19] Cobbold S Metcalfe S. Monoclonal antibodies that define canine homologues of human CD antigens: summary of the first international canine leukocyte antigen workshop (CLAW). Tissue Antigens. (1994) 43:137–54. doi: 10.1111/j.1399-0039.1994.tb02315.x, PMID: 8091414

[20] Holznagel E Hofmann-Lehmann R Leutenegger CM Allenspach K Huettner S Forster U. The role of in vitro-induced lymphocyte apoptosis in feline immunodeficiency virus infection: correlation with different markers of disease progression. J Virol. (1998) 72:9025–33. doi: 10.1128/JVI.72.11.9025-9033.1998, PMID: 9765447 PMC110319

[21] Krentz D Zenger K Alberer M Felten S Bergmann M Dorsch R. Curing cats with feline infectious peritonitis with an oral multi-component drug containing GS-441524. Viruses. (2021) 13:2228. doi: 10.3390/v13112228, PMID: 34835034 PMC8621566

[22] Meli ML Spiri AM Zwicklbauer K Krentz D Felten S Bergmann M. Fecal feline coronavirus RNA shedding and spike gene mutations in cats with feline infectious peritonitis treated with GS-441524. Viruses. (2022) 14:1069. doi: 10.3390/v14051069, PMID: 35632813 PMC9147249

[23] Zwicklbauer K Krentz D Bergmann M Felten S Dorsch R Fischer A. Long-term follow-up of cats in complete remission after treatment of feline infectious peritonitis with oral GS-441524. J Feline Med Surg. (2023) 25:25. doi: 10.1177/1098612X231183250, PMID: 37548535 PMC10811998

[24] Funder DC Ozer DJ. Evaluating effect size in psychological research: sense and nonsense. Adv Methods Pract Psychol Sci. (2019) 2:156–68. doi: 10.1177/2515245919847202

[25] Kipar A May H Menger S Weber M Leukert W Reinacher M. Morphologic features and development of granulomatous vasculitis in feline infectious peritonitis. Vet Pathol. (2005) 42:321–30. doi: 10.1354/vp.42-3-321 15872378

[26] Nishimura Y Shimojima M Sato E Izumiya Y Tohya Y Mikami T. Downmodulation of CD3ε expression in CD8α + β − T cells of feline immunodeficiency virus-infected cats. J Gen Virol. (2004) 85:2585–9. doi: 10.1099/vir.0.80102-0 15302952

[27] Rütgen BC Baszler E Weingand N Wolfesberger B Baumgartner D Hammer SE. Composition of lymphocyte subpopulations in normal and mildly reactive peripheral lymph nodes in cats. J Feline Med Surg. (2022) 24:77–90. doi: 10.1177/1098612X211005310, PMID: 33908810 PMC10812179

[28] Ackley CD Cooper MD. Characterization of a feline T-cell-specific monoclonal antibody reactive with a CD5-like molecule. Am J Vet Res. (1992) 53:466–71. doi: 10.2460/ajvr.1991.53.04.466, PMID: 1375007

[29] de Ruiter K van Staveren S Hilvering B Knol E Vrisekoop N Koenderman L. A field-applicable method for flow cytometric analysis of granulocyte activation: cryopreservation of fixed granulocytes. Cytometry A. (2018) 93:540–7. doi: 10.1002/cyto.a.23354, PMID: 29533506

[30] Maecker HT McCoy JP Jr FOCIS Human Immunophenotyping Consortium Amos M Elliott J Gaigalas A. A model for harmonizing flow cytometry in clinical trials. Nat Immunol. (2010) 11:975–8. doi: 10.1038/ni1110-975, PMID: 20959798 PMC3400260

[31] Byrne KM Kim HW Chew BP Reinhart GA Hayek MG. A standardized gating technique for the generation of flow cytometry data for normal canine and normal feline blood lymphocytes. Vet Immunol Immunopathol. (2000) 73:167–82. doi: 10.1016/s0165-2427(99)00163-4, PMID: 10690932

[32] Walker C Canfield PJ Love DN. Analysis of leucocytes and lymphocyte subsets for different clinical stages of naturally acquired feline immunodeficiency virus infection. Vet Immunol Immunopathol. (1994) 44:1–12. doi: 10.1016/0165-2427(94)90165-1, PMID: 7725628

[33] Roosje PJ van Kooten PJS Thepen T Bihari IC Rutten VPMG Koeman JP. Increased numbers of CD4+ and CD8+ T cells in lesional skin of cats with allergic dermatitis. Vet Pathol. (1998) 35:268–73. doi: 10.1177/030098589803500405, PMID: 9684970

[34] Dean GA Quackenbush SL Ackley CD Cooper MD Hoover EA. Flow cytometric analysis of T-lymphocyte subsets in cats. Vet Immunol Immunopathol. (1991) 28:327–35. doi: 10.1016/0165-2427(91)90124-u, PMID: 1683049

[35] Rabiger FV Rothe K von Buttlar H Bismarck D Büttner M Moore PF. Distinct features of canine non-conventional CD4−CD8α− double-negative TCRαβ+ vs. TCRγδ+ T cells. Front Immunol. (2019) 10:2748. doi: 10.3389/fimmu.2019.02748. eCollection 2019, PMID: 31824515 PMC6883510

[36] Yang H Parkhouse RME. Differential expression of CD8 epitopes amongst porcine CD8-positive functional lymphocyte subsets. Immunology. (1997) 92:45–52. doi: 10.1046/j.1365-2567.1997.00308.x, PMID: 9370923 PMC1363980

[37] Addison EG North J Bakhsh I Marden C Haq S al-Sarraj S. Ligation of CD8α on human natural killer cells prevents activation-induced apoptosis and enhances cytolytic activity. Immunology. (2005) 116:354–61. doi: 10.1111/j.1365-2567.2005.02235.x, PMID: 16236125 PMC1802415

[38] Trinchieri G. Biology of natural killer cells. Adv Immunol. (1989) 47:187–376. doi: 10.1016/S0065-2776(08)60664-1 2683611 PMC7131425

[39] Boysen P Gunnes G Pende D Valheim M Storset AK. Natural killer cells in lymph nodes of healthy calves express CD16 and show both cytotoxic and cytokine-producing properties. Dev Comp Immunol. (2008) 32:773–83. doi: 10.1016/j.dci.2007.11.006, PMID: 18177938

[40] Ferlazzo G Thomas D Lin SL Goodman K Morandi B Muller WA. The abundant NK cells in human secondary lymphoid tissues require activation to express killer cell Ig-like receptors and become cytolytic. J Immunol. (2004) 172:1455–62. doi: 10.4049/jimmunol.172.3.1455 14734722

[41] Galler A Rütgen BC Haas E Saalmüller A Hirt RA Gerner W. Immunophenotype of peripheral blood lymphocytes in dogs with inflammatory bowel disease. J Vet Intern Med. (2017) 31:1730–9. doi: 10.1111/jvim.14812, PMID: 28862348 PMC5697185

[42] Vermeulen BL Devriendt B Olyslaegers DA Dedeurwaerder A Desmarets LM Grauwet KL. Natural killer cells: frequency, phenotype and function in healthy cats. Vet Immunol Immunopathol. (2012) 150:69–78. doi: 10.1016/j.vetimm.2012.08.010, PMID: 22985632

[43] Moore PF Rossitto PV Danilenko DM Wielenga JJ Raff RF Severns E. Monoclonal antibodies specific for canine CD4 and CD8 define functional T-lymphocyte subsets and high-density expression of CD4 by canine neutrophils. Tissue Antigens. (1992) 40:75–85. doi: 10.1111/j.1399-0039.1992.tb01963.x, PMID: 1412420

[44] Dean GA Reubel GH Moore PF Pedersen NC. Proviral burden and infection kinetics of feline immunodeficiency virus in lymphocyte subsets of blood and lymph node. J Virol. (1996) 70:5165–9. doi: 10.1128/JVI.70.8.5165-5169.1996, PMID: 8764024 PMC190471

[45] Tedder TF Clement LT Cooper MD. Expression of C3d receptors during human B cell differentiation: immunofluorescence analysis with the HB-5 monoclonal antibody. J Immunol. (1984) 133:678–83. doi: 10.4049/jimmunol.133.2.678 6234356

[46] Takano T Azuma N Hashida Y Satoh R Hohdatsu T. B-cell activation in cats with feline infectious peritonitis (FIP) by FIP-virus-induced B-cell differentiation/survival factors. Arch Virol. (2009) 154:27–35. doi: 10.1007/s00705-008-0265-9, PMID: 19043660 PMC7087278

[47] Chang WL Gonzalez DF Kieu HT Castillo LD Messaoudi I Shen X. Changes in circulating B cell subsets associated with aging and acute SIV infection in Rhesus macaques. PLoS One. (2017) 12:e0170154. doi: 10.1371/journal.pone.0170154. PMID: 28095513, PMID: 28095513 PMC5240950

[48] Ziegler-Heitbrock L. Monocyte subsets in man and other species. Cell Immunol. (2014) 289:135–9. doi: 10.1016/j.cellimm.2014.03.019 24791698

[49] Rzepecka A Żmigrodzka M Witkowska-Piłaszewicz O Cywińska A Winnicka A. CD4 and MHCII phenotypic variability of peripheral blood monocytes in dogs. PLoS One. (2019) 14:e0219214. doi: 10.1371/journal.pone.0219214, PMID: 31269060 PMC6608971

[50] Martini V Bernardi S Giordano A Comazzi S. Flow cytometry expression pattern of CD44 and CD18 markers on feline leukocytes. J Vet Diagn Invest. (2020) 32:706–9. doi: 10.1177/1040638720945670, PMID: 32718218 PMC7488972

[51] Goldmann F Bauer N Moritz A. Evaluation of the IDEXX ProCyte dx analyzer for dogs and cats compared to the Siemens ADVIA 2120 and manual differential. Comp Clin Path. (2014) 23:283–96. doi: 10.1007/s00580-012-1608-1

[52] Pedersen NC. A review of feline infectious peritonitis virus infection: 1963-2008. J Feline Med Surg. (2009) 11:225–58. doi: 10.1016/j.jfms.2008.09.008, PMID: 19254859 PMC7129802

[53] Saverio P Alessia G Vito T Stefano G. Critical assessment of the diagnostic value of feline alpha1-acid glycoprotein for feline infectious peritonitis using the likelihood ratios approach. J Vet Diagn Invest. (2007) 19:266–72. doi: 10.1177/104063870701900306, PMID: 17459855

[54] Tecles F Caldín M Tvarijonaviciute A Escribano D Martínez-Subiela S Cerón JJ. Serum biomarkers of oxidative stress in cats with feline infectious peritonitis. Res Vet Sci. (2015) 100:12–7. doi: 10.1016/j.rvsc.2015.02.007, PMID: 25819115 PMC7111829

[55] Hazuchova K Held S Neiger R. Usefulness of acute phase proteins in differentiating between feline infectious peritonitis and other diseases in cats with body cavity effusions. J Feline Med Surg. (2017) 19:809–16. doi: 10.1177/1098612X16658925, PMID: 27432437 PMC11104113

[56] Sasaki K Ma Z Khatlani TS Okuda M Inokuma H Onishi T. Evaluation of feline serum amyloid a (SAA) as an inflammatory marker. J Vet Med Sci. (2003) 65:545–8. doi: 10.1292/jvms.65.545, PMID: 12736442

[57] Giordano A Spagnolo V Colombo A Paltrinieri S. Changes in some acute phase protein and immunoglobulin concentrations in cats affected by feline infectious peritonitis or exposed to feline coronavirus infection. Vet J. (2004) 167:38–44. doi: 10.1016/s1090-0233(03)00055-8, PMID: 14623149 PMC7128346

[58] Riond B Waßmuth AK Hartnack S Hofmann-Lehmann R Lutz H. Study on the kinetics and influence of feline platelet aggregation and deaggregation. BMC Vet Res. (2015) 11:276. doi: 10.1186/s12917-015-0590-7, PMID: 26542105 PMC4635602

