# Supplementary material for: Adapting the SMART tube technology for flow cytometry in feline full blood samples
Source: Front Vet Sci. 2024 Jun 26;11:1377414. doi: 10.3389/fvets.2024.1377414 (PMC11234156; doi:10.3389/fvets.2024.1377414)
Supplement: Supplementary file 1 [file Table_1.docx]

Supplementary Material

# Supplementary Table S1. Storage time at -80°C for SMT samples used in this study.

|  | | |
| --- | --- | --- |
| **Sample type** | | **Storage duration in days (mean)** |
| Validation of antibody panel and gating strategy | | 7–354 (254) |
| Validation of the new SMT technique | | 2–4 (2) |
| Clinical application for long-term storage under acute disease conditions (cats with FIP) | Day 0 | 825–873 (859) |
|  | Day 7 | 818–866 (852) |
|  | Day 14 | 845–894 (884) |
|  | Day 28 | 830–881 (871) |
|  | Day 56 | 803–853 (843) |
|  | Day 83 | 742–826 (783) |
| Clinical application for long-term storage under healthy conditions (control cats) | | 743–890 (844) |
| FIP=feline infectious peritonitis, SMT=SMART Tubes analyzed with flow cytometry | | |

# Supplementary Table S2. Absolute cell counts in cells x10^9^/l obtained by an automatic hematology analyzer (Diff) or flow cytometry of smart tube (SMT) and fresh blood (FB) samples of 20 healthy cats.

|  | **Neutrophils** | | | **Monocytes** | | | **Lymphocytes**^*^ | | |
| --- | --- | --- | --- | --- | --- | --- | --- | --- | --- |
| **Cat ID** | **Diff** | **SMT** | **FB** | **Diff** | **SMT** | **FB** | **Diff** | **SMT** | **FB** |
| 1 | 8.93 | 8.73 | 6.94 | 0.34 | 0.32 | 0.33 | 2.86 | 2.79 | 3.08 |
| 2 | 8.13 | 7.48 | 6.62 | 0.42 | 0.45 | 0.44 | 5.18 | 4.24 | 4.33 |
| 3 | 6.80 | 6.31 | 6.59 | 0.97 | 0.98 | 0.98 | 6.50 | 6.16 | 5.97 |
| 4 | 6.73 | 6.85 | 6.78 | 0.26 | 0.27 | 0.27 | 1.48 | 1.19 | 1.19 |
| 5 | 3.89 | 3.31 | 2.65 | 0.24 | 0.22 | 0.22 | 6.03 | 5.86 | 5.99 |
| 6 | 11.29 | 11.65 | 12.09 | 0.27 | 0.23 | 0.25 | 3.02 | 2.46 | 2.51 |
| 7 | 16.09 | 16.03 | 16.20 | 0.37 | 0.33 | 0.31 | 1.82 | 1.58 | 1.60 |
| 8 | 3.68 | 4.17 | 4.22 | 0.78 | 0.67 | 0.67 | 2.68 | 2.18 | 2.20 |
| 9 | 5.53 | 5.40 | 5.41 | 0.48 | 0.42 | 0.48 | 1.03 | 0.94 | 0.97 |
| 10 | 14.82 | 15.54 | 15.58 | 0.63 | 0.63 | 0.55 | 3.91 | 3.20 | 3.23 |
| 11 | 3.51 | 3.40 | 3.34 | 0.31 | 0.28 | 0.28 | 1.87 | 2.13 | 2.20 |
| 12 | 5.07 | 4.79 | 5.02 | 0.29 | 0.31 | 0.28 | 6.05 | 4.54 | 5.11 |
| 13 | 3.23 | 3.28 | 3.00 | 0.31 | 0.31 | 0.30 | 9.88 | 9.61 | 10.00 |
| 14 | 5.37 | 6.16 | 6.13 | 0.66 | 0.60 | 0.61 | 3.02 | 2.50 | 2.54 |
| 15 | 10.01 | 9.90 | 9.40 | 0.52 | 0.51 | 0.54 | 1.38 | 1.23 | 1.21 |
| 16 | 13.84 | 15.13 | 14.42 | 0.58 | 0.57 | 0.51 | 3.40 | 1.85 | 1.82 |
| 17 | 8.67 | 8.71 | 8.19 | 0.79 | 0.82 | 0.86 | 2.65 | 2.30 | 2.29 |
| 18 | 7.16 | 6.81 | 6.99 | 0.29 | 0.32 | 0.31 | 1.03 | 0.84 | 0.84 |
| 19 | 3.40 | 3.34 | 2.93 | 0.27 | 0.25 | 0.29 | 3.13 | 3.07 | 3.43 |
| 20 | 4.57 | 4.48 | 4.35 | 0.20 | 0.20 | 0.20 | 4.63 | 4.36 | 4.49 |

^*^Lymphocyte numbers were calculated by adding up the numbers of CD4+, CD8+, CD21+ and marker negative lymphocytes

# Supplementary Table S3. Absolute cell counts in cells x10^9^/l obtained by an automatic hematology analyzer Diff/or flow cytometry of smart tube SMT samples of 5 cats with feline infectious peritonitis (FIP) and 5 healthy cats.

| Cats with FIP (Diff/SMT) | | | | | | |  |  |  |  |  |  |
| --- | --- | --- | --- | --- | --- | --- | --- | --- | --- | --- | --- | --- |
|  |  | #1 | #2 | #3 | #4 | #5 |  |  |  |  |  |  |
| Day 14 | Neutrophils | 8.33/8.01 | 8.19/7.95 | 5.62/5.23 | 5.35/4.88 | 7.14/7.40 |  |  |  |  |  |  |
|  | Monocytes | 0.39/0.37 | 0.61/0.57 | 0.50/0.56 | 0.59/0.52 | 0.35/0.32 |  |  |  |  |  |  |
|  | Lymphocytes^*^ | 8.19/7.60 | 7.81/7.32 | 7.72/7.21 | 3.56/3.00 | 4.28/3.88 |  |  |  |  |  |  |
| Day 28 | Neutrophils | 7.34/6.93 | 6.97/6.00 | 9.02/8.56 | 5.78/5.33 | 4.05/3.89 |  |  |  |  |  |  |
|  | Monocytes | 0.37/0.35 | 0.54/0.52 | 0.56/0.60 | 0.50/0.47 | 0.21/0.20 |  |  |  |  |  |  |
|  | Lymphocytes^*^ | 6.10/5.35 | 5.89/5.05 | 8.27/7.69 | 7.13/6.00 | 4.41/4.10 |  |  |  |  |  |  |
| Day 56 | Neutrophils | 6.13/5.78 | 6.13/6.34 | 7.90/7.43 | 5.72/6.20 | 5.11/4.49 |  |  |  |  |  |  |
|  | Monocytes | 0.25/0.27 | 0.34/0.30 | 0.32/0.29 | 0.48/0.50 | 0.20/0.18 |  |  |  |  |  |  |
|  | Lymphocytes^*^ | 6.75/5.60 | 8.27/7.99 | 7.73/6.80 | 8.84/8.34 | 3.44/3.27 |  |  |  |  |  |  |
| Day 83 | Neutrophils | 5.68/5.22 | 4.20/4.52 | 9.27/8.88 | 4.32/4.02 | 8.27/7.95 |  |  |  |  |  |  |
|  | Monocytes | 0.27/0.24 | 0.37/0.35 | 0.29/0.28 | 0.20/0.18 | 0.30/0.27 |  |  |  |  |  |  |
|  | Lymphocytes^*^ | 6.43/5.82 | 6.06/6.20 | 6.40/5.60 | 8.14/8.60 | 4.83/4.54 |  |  |  |  |  |  |
|  |  |  |  |  |  |  |  |  |  |  |  |  |
|  |  |  |  |  |  |  |  |  |  |  |  |  |
| Healthy control Cats (Diff/SMT) | | | | | | |  |  |  |  |  |  |
|  |  | #1 | #2 | #3 | #4 | #5 |  |  |  |  |  |  |
|  | Neutrophils | 2.74/3.00 | 4.22/3.99 | 3.58/3.12 | 1.97/1.69 | 4.06/3.59 |  |  |  |  |  |  |
|  | Monocytes | 0.32/0.29 | 0.18/0.20 | 0.12/0.13 | 0.10/0.09 | 0.30/0.28 |  |  |  |  |  |  |
|  | Lymphocytes^*^ | 4.35/4.01 | 2.75/2.45 | 3.35/2.88 | 5.77/5.20 | 3.01/2.82 |  |  |  |  |  |  |

Diff=differential blood count with automatic hemocytometer, FIP=feline infectious peritonitis, SMT=SMART Tubes analyzed with flow cytometry

^*^ all lymphocyte numbers were calculated by adding up the numbers of CD4+, CD8+, CD21+ and marker negative lymphocytes

# Supplementary Figure 1: Non-functional monoclonal antibodies

Staining of anti-CD4-FITC (clone 3-4F4) (A), anti-CD21-PerCP-Cy5.5 (clone CA2.1D6) (B) and anti-CD14-PacBlue (clone TÜK14) (C) on fresh blood (left) and fixed blood (right). Cells were gated for single leukocytes. Forward scatter/Side scatter (FSC/SSC) properties of positive populations were shown as overlays with total leukocytes.

# Supplementary Figure 2: Functional monoclonal antibodies

Representative staining of anti-CD4-FITC (clone vpg34) (A), anti-CD8-PE (clone fCD8) (B), anti-CD21-APC (clone B-ly4) (C), anti-MHCII-PacBlue (clone PF6J-6D) (D) on fresh blood (left) and fixed blood (right). FSC settings were adjusted to get comparable pictures. Cells were gated for single leukocytes. FSC/SSC properties of positive populations were shown as overlays with total leukocytes. Histograms show overlays of isotype control (dashed black lines) and the positive population from the dot blot on the left (blue). Mean fluorescence intensity (MFI/median) for each fluorochrome is given in each histogram.

# Supplementary Figure 3: Agreement Diff/FB/SMT

Agreement between the absolute cell numbers (x10^9^/l) for neutrophils, monocytes, and lymphocytes (A–C) of 20 healthy cats obtained with an automated hematology analyzer (Diff), by flow cytometry within 24h (FB) and with fixed/frozen Smart Tubes (SMTs) and agreement between lymphocyte subpopulations obtained by flow cytometry with FB and SMT (D). Bland-Altman diagrams show the differences (∆) between the two compared methods, plotted against the average of the two results. The mean difference (bias, dashed purple line with lower and upper CI limiting the purple area) between both methods and the limits of Agreement (LoA) are shown; upper LoA with dashed green line (with lower and upper CI limiting the green area) and lower LoA with dashed red line (with lower and upper CI limiting the red area.
