# Supplementary figures and images for: Adapting the SMART tube technology for flow cytometry in feline full blood samples
Source: Front Vet Sci. 2024 Jun 26;11:1377414. doi: 10.3389/fvets.2024.1377414 (PMC11234156; doi:10.3389/fvets.2024.1377414)

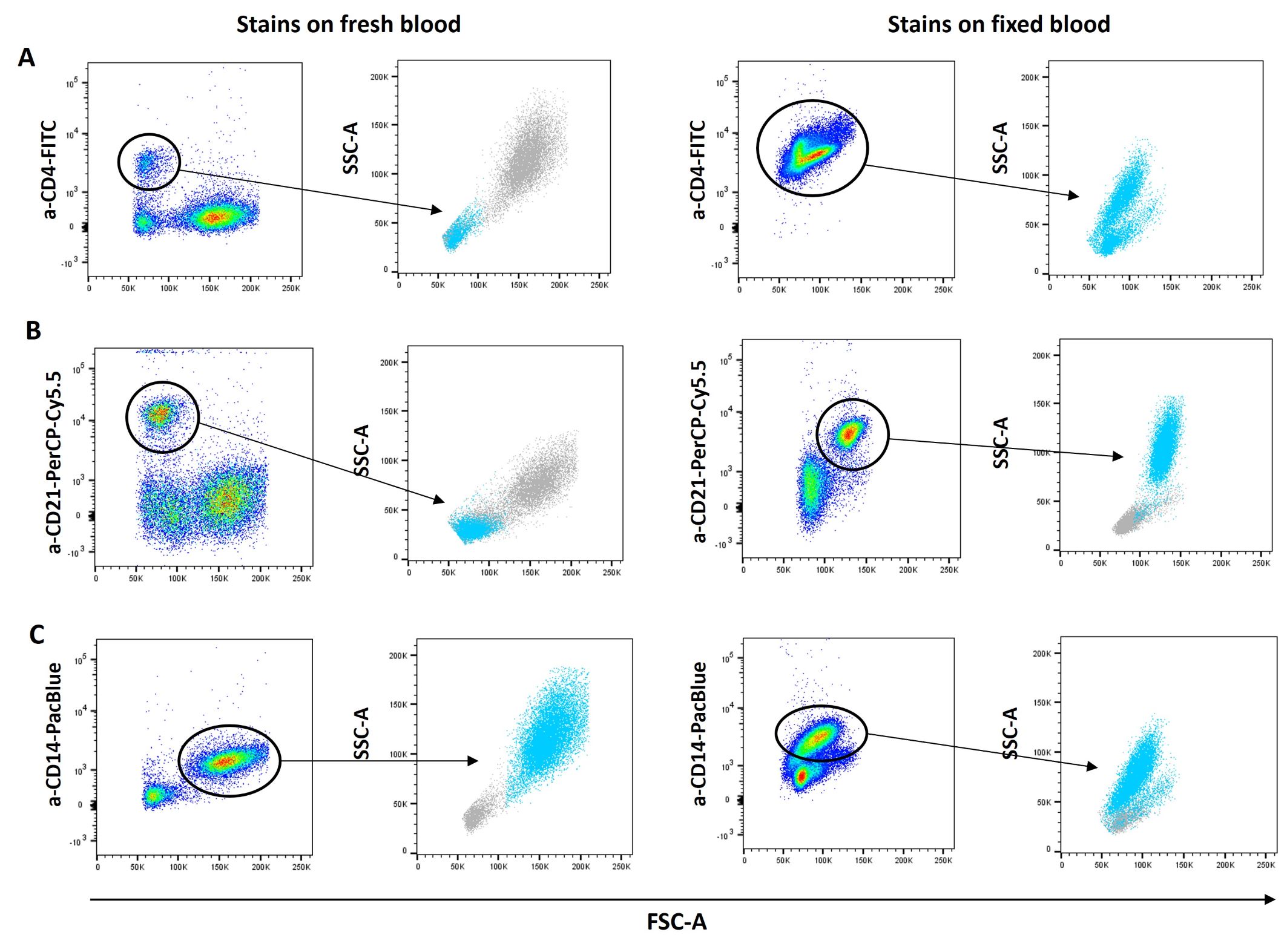

Supplement: Supplementary file 2 [file Image_1.JPEG]

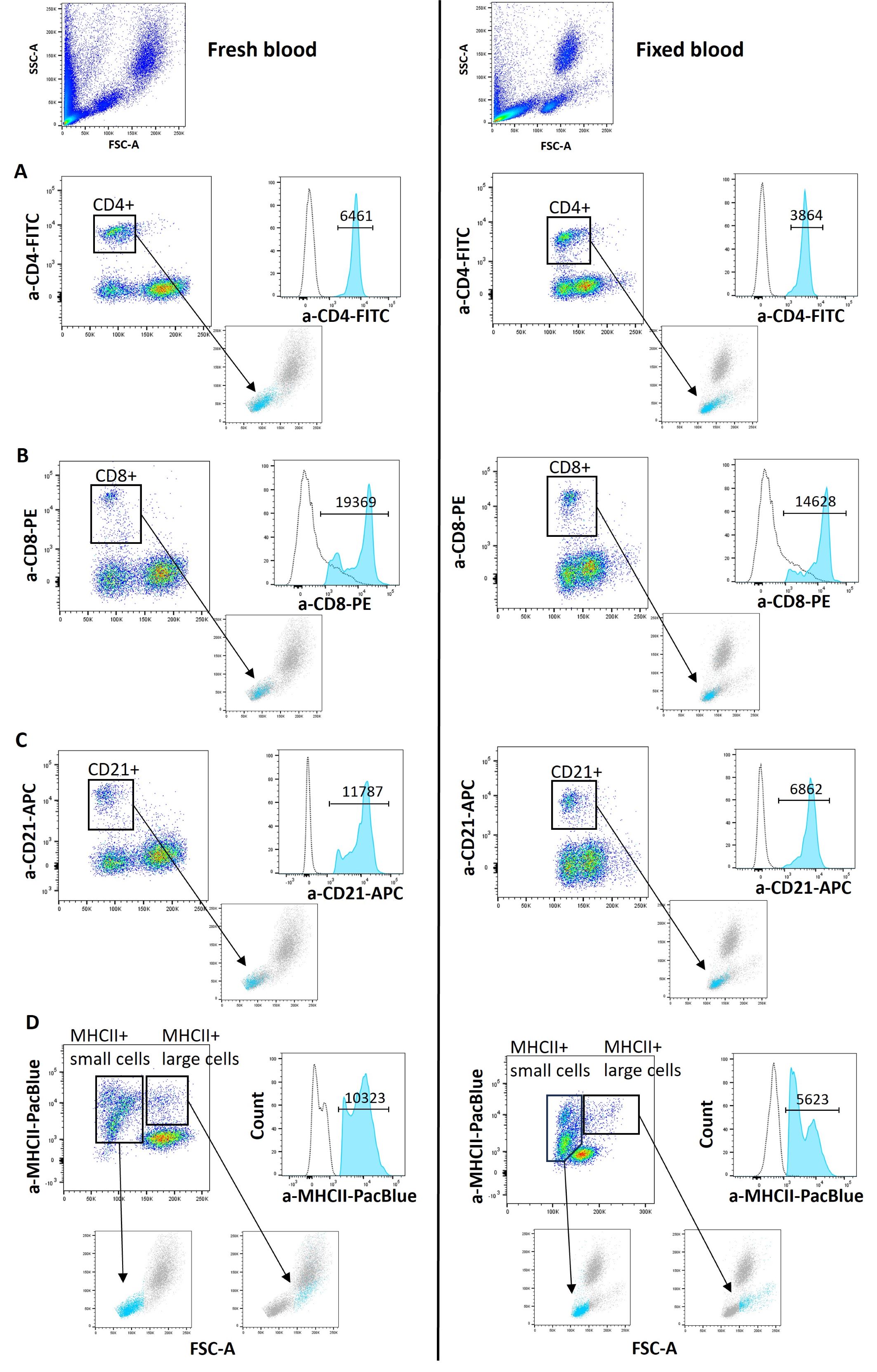

Supplement: Supplementary file 3 [file Image_2.JPEG]

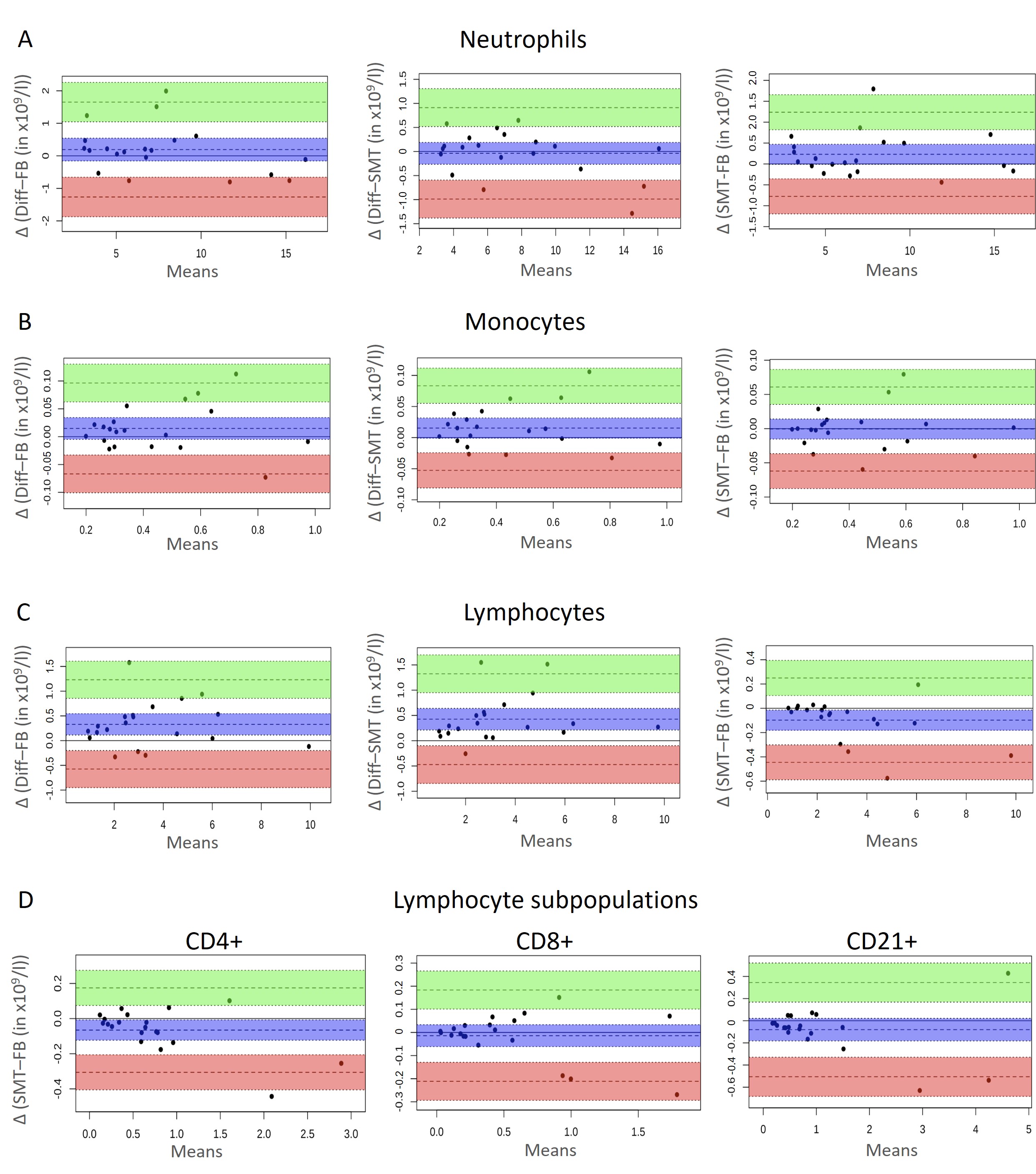

Supplement: Supplementary file 4 [file Image_3.JPEG]
